# A Novel Framework to Assess Clinical Information in Digital Health Technologies: Cross-Sectional Survey Study

**DOI:** 10.2196/58125

**Published:** 2025-05-30

**Authors:** Kayode Philip Fadahunsi, Petra A Wark, Nikolaos Mastellos, Ana Luisa Neves, Joseph Gallagher, Azeem Majeed, Josip Car

**Affiliations:** 1 Department of Primary Care and Public Health, Imperial College London, White City Campus, 80–92 Wood Lane, London, W12 0BZ, United Kingdom, +44 02075943368; 2 Centre for Healthcare and Communities, Coventry University, Richard Crossman, Priory Street, Coventry, CV1 5FB, United Kingdom; 3 Global Health Research Group, School of Medicine, University College Dublin, Dublin, Ireland; 4 School of Life Course and Population Sciences, King’s College London, London, United Kingdom

**Keywords:** information quality, digital health technology, patient safety, structural equation modeling, reliability, information, applicability, consistency, validity, Clinical Information Quality, CLIQ, cross-sectional, survey, United Kingdom, questionnaire, health care professional, doctor, nurse, practitioner, pragmatic, health care setting

## Abstract

**Background:**

Digital health is a critical driver of quality, safety, and efficiency in health care. However, poor quality of clinical information in digital health technologies (DHTs) can compromise the quality and safety of care. The Clinical Information Quality (CLIQ) framework was developed, based on a systemic review of literature and an international eDelphi study, as a tool to assess the quality of clinical information in DHTs.

**Objectives:**

The aim of this study is to assess the applicability, internal consistency, and construct validity of the CLIQ framework.

**Methods:**

This study was conducted as a cross-sectional survey of health care professionals across the United Kingdom who regularly use SystmOne electronic health records. Participants were invited through emails and social media platforms. The CLIQ questionnaire was administered as a web-based survey. Spearman correlation coefficients were computed to investigate the linear relationship between the dimensions in the CLIQ framework. The Cronbach α coefficients were computed to assess the internal consistency of the global scale (ie, CLIQ framework) and the subscales (ie, the informativeness, availability, and usability categories). Confirmatory factor analysis was used to assess the extent to which the survey data supported the construct validity of the CLIQ framework.

**Results:**

A total of 109 health care professionals completed the survey, of which two-thirds (67, 61.5%) were doctors and a quarter (26, 23.9%) were nurses or advanced nurse practitioners. Overall, the CLIQ dimensions had good quality scores except for portability, which had a modest score. The inter-item correlations were all positive and not likely due to chance. The Cronbach α coefficient for the overall CLIQ framework was 0.89 (95% CI 0.85‐0.92). The confirmatory factor analysis provided a modest support for the construct validity of the CLIQ framework with the comparative fit index of 0.86 and standardized root mean square residual of 0.08.

**Conclusions:**

The CLIQ framework demonstrated a high reliability and a modest construct validity. The CLIQ framework offers a pragmatic approach to assessing the quality of clinical information in DHTs and could be applied as part of information quality assurance systems in health care settings to improve quality of health information.

## Introduction

Digital health is critical to quality, safety, and efficiency of health care services [1]. Digital health technologies (DHTs) can enhance the delivery of health care services in several ways [2]. Electronic health records (EHRs) make medical records readily available at the point of care [3]. Electronic prescribing systems reduce the incidents of medication errors [4]. Clinical decision support systems support clinicians in decision-making [5,6]. Mobile health (mHealth) apps support self-management of chronic diseases [7].

However, poor quality of clinical information in DHTs can compromise the quality and safety of care [8,9]. A systematic review of literature reported widespread incidents of delayed, missing, partial, and wrong information in DHTs resulting in adverse outcomes and deaths [10]. Most of the information quality problems reported in the included studies were based on incidents reporting systems [10]. While retrospective lessons based on adverse events in the incidents reporting systems could be useful, it is more important to identify and address information quality problems as a proactive measure to prevent adverse events, hence the need for an information quality framework.

In another systematic review [11], we identified 10 existing information quality frameworks for DHTs comprising 5 frameworks for EHRs [12-16] and one each for clinical decision support systems [17], cloud-based information systems [18], electronic medical records [19], mobile and web-based telemedicine apps [20], and primary care databases [13]. Although these frameworks identified dimensions that are relevant to assessing clinical information in DHTs, most were developed without inputs of clinicians and did not provide a tool that can be used to assess DHTs [11]. To overcome these limitations, we developed the Clinical Information Quality (CLIQ) framework for DHTs.

The CLIQ framework identifies, defines, and integrates 14 dimensions that are relevant to assessing clinical information in DHTs [11,21]. The dimensions in the CLIQ framework are grouped into 3 categories—informativeness, availability, and usability. The informativeness category relates to the usefulness of clinical information in patient care. Dimensions in the informativeness category include accuracy, completeness, interpretability, plausibility, relevance, and trustworthiness. The availability category relates to the functionality of the DHTs holding clinical information. Dimensions in this category include accessibility, portability, searchability, security, and timeliness. The usability category—comprising conformance, consistency of presentation, and maintainability—concerns the ease of use of clinical information. The definitions of the dimensions in the CLIQ framework are shown in Table 1.

**Figure 1.**
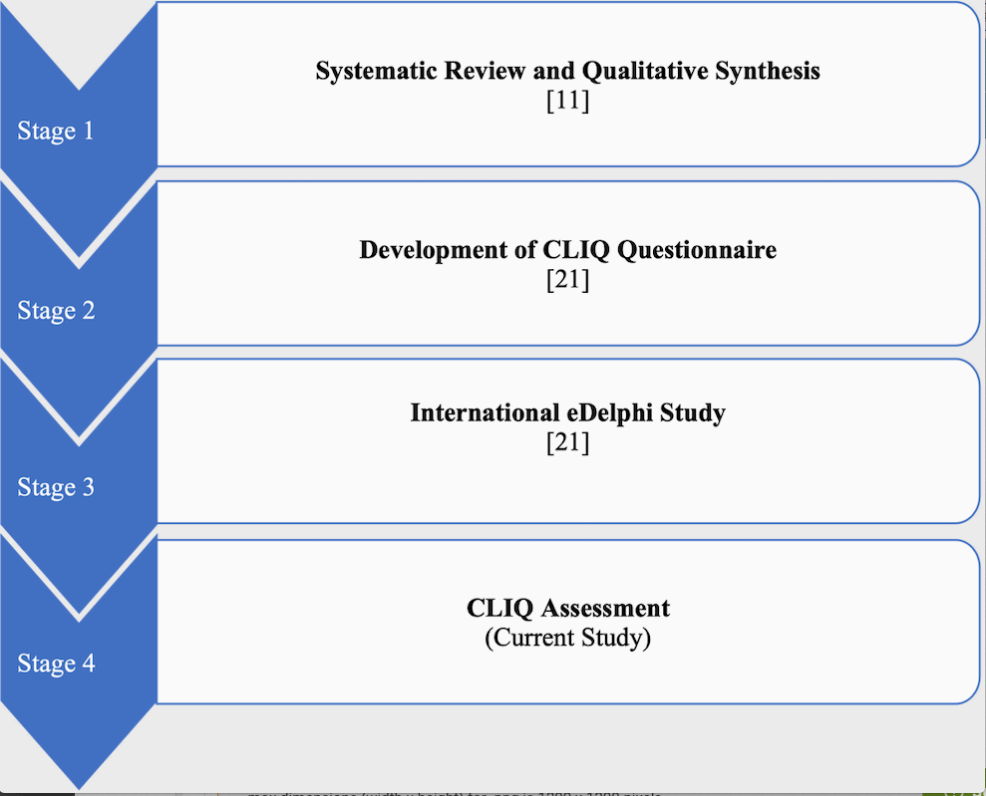
Stages of CLIQ framework Development.CLIQ: Clinical Information Quality.

The CLIQ framework was developed in 4 successive stages as shown in Figure 1. An initial CLIQ framework was developed through a systematic review and qualitative synthesis of existing information quality frameworks for DHTs [11]. A CLIQ assessment questionnaire was then developed based on the CLIQ framework and further evidence from literature [21]. The questionnaire offers a pragmatic approach to assessing clinical information in DHTs based on relatable clinical scenarios. The framework and the accompanying questionnaire were revised via an international eDelphi study among 35 clinicians from 10 different countries [21].

The development and modification of the CLIQ framework through a systematic review of literature and an international eDelphi study resulted in an evidence-based and user-friendly framework that is grounded in the literature and the views of clinicians who are users of clinical information. However, these approaches addressed only the content and face validity of the CLIQ framework and not its applicability, reliability, and construct validity. Therefore, the aim of this study is to assess the applicability, internal consistency, and construct validity of the CLIQ framework.

**Table 1.** Clinical Information quality framework for digital health technologies.

Clinical information quality dimensions and their definition	
Informativeness: the usefulness of digital information for clinical purposes	
Accuracy	The extent to which information is accurate.
Completeness	The extent to which no required information is missing.
Interpretability	The extent to which information can be interpreted.
Plausibility	The extent to which information makes sense based on clinical knowledge.
Trustworthiness	The extent to which the source of information is trustworthy and verifiable.
Relevance	The extent to which information is useful for patient care.
Availability: the functionality of the system holding clinical information	
Accessibility	The extent to which information is accessible.
Portability	The extent to which information can be moved or transferred between different systems.
Searchability	The extent to which needed information can be found.
Security	The extent to which information is protected from unauthorized access, corruption, and damage.
Timeliness	The extent to which information is up to date.
Usability: the ease of use of clinical information	
Conformance	The extent to which information is presented in a format that complies with institutional, national, or international standards.
Consistency of presentation	The extent to which presentation of information adheres to the same set of institutional, national, or international standards.
Maintainability	The extent to which information can be maintained (eg, modified, corrected, updated, adapted, and upgraded) to achieve intended improvement.

## Methods

### Study Design

This study was conducted as a web-based cross-sectional survey of health care professionals across the United Kingdom who use SystmOne EHRs (The Phoenix Partnership Ltd). The cross-sectional survey approach allowed the assessment of the applicability, internal consistency, and construct validity of the CLIQ framework. The web-based approach offered a convenient, affordable, and pragmatic way of conducting a study and collecting data. The choice of SystmOne was informed by its wide use across different health care settings in the United Kingdom, including general practices, urgent care centers, social care services, hospitals, and prison medical services to manage about 61 million EHRs [22]. CHERRIES (Checklist for Reporting Results of Internet E-Surveys) was used to guide the survey report [23].

### Study Participants

Eligibility criteria for the study included being a health care professional with a clinical role and a regular user of SystmOne (defined as using SystmOne as part of routine professional activities to document clinical information). Administrative staff using SystmOne and health care professionals using SystmOne occasionally (eg, to check clinical records) were not eligible to participate in the study. Participants were invited through emails and social media platforms, including Facebook, LinkedIn, WhatsApp, Yammer, Digital Health Networks, and the Future National Health System Collaboration platform. Although there is no consensus about the adequate sample size needed to validate a questionnaire, the literature recommends recruiting at least 10 participants for each item of the scale being validated as a rule of thumb [24]. As there are 14 dimensions of the CLIQ framework, this was equivalent to 140 participants in our study.

### Data Collection

Health care professionals were invited to use the CLIQ framework to evaluate the information quality of SystmOne. The survey questionnaire comprised the 14 items of the CLIQ framework and 2 questions about the occupation of the respondents to assess their eligibility (Multimedia Appendix 1). Each question was displayed on a separate mobile or computer screen to the participants. The responses were made mandatory to avoid missing data, which could limit the assessment of the construct validity [25]. Participants were able to change their answers using the back button. Bot detection and prevention of multiple submissions were turned on in Qualtrics (Qualtrics). An invitation containing a link to the participant information sheet, the consent form, and the questionnaire was shared with the health care professionals electronically through the channels described earlier. Two reminders, at least 2 weeks apart, were sent to encourage participation. Health care professionals were also encouraged to share the invitation with colleagues. Data collection took place between February 27 and June 7, 2022. The questionnaire was administered through a web-based survey platform, Qualtrics.

### Data Analysis

The survey result was downloaded from Qualtrics in an excel format and imported into SPSS (version 20; IBM Statistics). Only completed entries were analyzed as uncompleted entries stopped at the consent. SPSS was used to conduct descriptive statistical analyses and compute correlation coefficients and Cronbach α scores.

The 3 options for each dimension (eg, accurate, partly accurate, and not accurate) were recoded into the integers 1, 2, and 3, representing low, modest, and good quality, respectively. A descriptive statistical analysis was conducted and interpreted to demonstrate the applicability of the CLIQ framework. The distribution of the responses was expressed as frequencies, percentages, means, and SDs.

Spearman correlation coefficients were computed to investigate the linear relationship between the dimensions in the CLIQ framework. The ordinal nature of the data informed the choice of Spearman coefficients. Correlation coefficients of 0.1‐0.2 were regarded as poor, 0.3‐0.5 as fair, 0.6‐0.7 as moderate, and 0.8‐0.9 as very strong [26] .

The Cronbach α coefficient was computed to assess the internal consistency of the global scale (ie, CLIQ framework) and the subscales (ie, the informativeness, availability, and usability categories). A Cronbach α coefficient of 0.7 or above was regarded as an indication of the reliability of the scale, and an alpha coefficient between 0.6 and 0.7 was considered acceptable [26].

The SPSS data file was subsequently exported to SPSS Amos (version 28; IBM Statistics) to assess the construct validity of the CLIQ framework. Confirmatory factor analysis, a structural equation modeling technique, was used to assess the extent to which the survey data supported the construct validity of the CLIQ framework [27]. Confirmatory factor analysis was adopted because the CLIQ framework has multiple subscales (ie, informativeness, availability, and usability categories) that were predetermined [11].

The maximum likelihood estimation method was used for the confirmatory factor analysis. The model fit was assessed based on the Standardized Root Mean Square Residual (SRMR) and Comparative Fit Index (CFI) as recommended in the literature for studies with a sample size of less than 250 [28]. A CFI greater than 0.9 and an SRMR less than 0.08 indicate model fit [28].

### Ethical Considerations

Informed consent was obtained from each participant at the beginning of the web-based survey after they had been provided with the participant information sheet, containing detailed information about the study objectives, expectation of the participants, duties of the researchers, and relevant contacts. Participation was voluntary. A secure web-based survey platform was used for data collection as already described. No personal information that could be used to identify the participants was collected in the survey. Survey responses were anonymized with codes automatically assigned to participants by the survey platform. The results were securely stored in Imperial College Shared Drive. Participants could withdraw from the study at any time without giving any reasons. However, once the survey was submitted, the data could not be withdrawn as the responses were anonymous. Participants were not compensated for taking part. Ethical approval was obtained from the Imperial College research ethics committee (21IC7415).

## Results

### Participants’ Characteristics

A total of 109 health care professionals completed the survey, with two-thirds (67, 61.5%) being doctors and almost a quarter (26, 23.9%) being nurses or advanced nurse practitioners. The rest of the participants had other clinical occupations. Table 2 shows the distribution of the participants by occupation.

**Table 2.** Distribution of the occupation of the survey participants (N=109).

Occupation	Values, n (%)
Doctors	67 (61.5)
Nurses and advanced nurse practitioners	26 (23.9)
Health care assistants	5 (4.6)
Physiotherapists and occupational therapists	4 (3.7)
Pharmacists	2 (1.8)
Podiatrists	2 (1.8)
Physician associates	1 (0.9)
Therapy support workers	1 (0.9)
Community health workers	1 (0.9)

### Participants’ Assessment of Quality of the Dimensions

The mean quality score assigned to each dimension ranged from 2.2 for portability to 2.9 for security of clinical information in SystmOne (1, 2, and 3 indicate low, modest, and good quality, respectively). Most participants (97/109, 89%) ranked security of clinical information in SystmOne as good while only more than a third of the participants (42/109, 38.5%) ranked portability of clinical information in SystmOne as good. The summary of the assessment result is shown in Table 3.

**Table 3.** Clinical Information Quality of SystmOne as assessed by the participants.

Dimension	Assessment of the quality of the dimension, n (%)			Mean score (SD)
	Good	Modest	Low	
Accuracy	73 (67)	32 (29.4)	4 (3.7)	2.6 (0.6)
Completeness	63 (57.8)	43 (39.4)	3 (2.8)	2.6 (0.6)
Interpretability	82 (75.2)	26 (23.9)	1 (0.9)	2.7 (0.5)
Plausibility	92 (84.4)	17 (15.6)	0	2.8 (0.4)
Relevance	89 (81.7)	20 (18.3)	0	2.8 (0.4)
Trustworthiness	94 (86.2)	15 (13.8)	0	2.9 (0.3)
Accessibility	77 (70.6)	31 (28.4)	1 (0.9)	2.7 (0.5)
Portability	42 (38.5)	49 (45)	18 (16.5)	2.2 (0.7)
Searchability	56 (51.4)	49 (45)	4 (3.7)	2.4 (0.6)
Security	97 (89)	12 (11)	0	2.9 (0.3)
Timeliness	71 (65.1)	35 (32.1)	3 (2.8)	2.6 (0.5)
Conformance	77 (70.6)	27 (24.8)	5 (4.6)	2.7 (0.6)
Consistency of presentation	77 (70.6)	22 (20.2)	10 (9.2)	2.6 (0.7)
Maintainability	70 (64.2)	33 (30.3)	6 (5.5)	2.6 (0.6)

### Inter-item Correlation

There were positive correlations between all possible pairs of dimensions in the CLIQ framework (Multimedia Appendix 2). All the correlation coefficients were statistically significant (represented as asterisks) except for the correlations of portability with each of plausibility, relevance, trustworthiness, and security. There is a strong statistically significant correlation between conformance and consistency (Spearman ρ=0.751; *P*<.001).

### Internal Consistency of the CLIQ Framework

The Cronbach α coefficients and corresponding 95% CIs for the informativeness, availability, and usability subscales were 0.78 (95% CI 0.70‐0.84), 0.69 (95% CI 0.58‐0.77), and 0.83 (95% CI 0.77‐0.88), respectively. Once security was removed from the availability subscale, the Cronbach α coefficient for the availability subscale increased marginally to 0.70 (95% CI 0.60‐0.78). The Cronbach α coefficient for the overall CLIQ framework is 0.89 (95% CI 0.85‐0.92).

### Construct Validity of the CLIQ Framework

Although the chi-square goodness-of-fit test (*χ*^2^_2.10_=155.69; *P*<.001) did not demonstrate fitness of the model, CFI (0.86) and SRMR (0.08) suggest that the model fits modestly with the data. All the factor loadings (ie, covariance estimates) were positive and statistically significant, as shown in Table 4. Significant tests (SE, critical ratio, and *P* value) were not reported for the first item in each category (ie, accuracy, accessibility, and conformance) because their unstandardized covariance values were fixed as 1, which is a standard procedure in confirmatory factor analysis.

**Figure 2.**
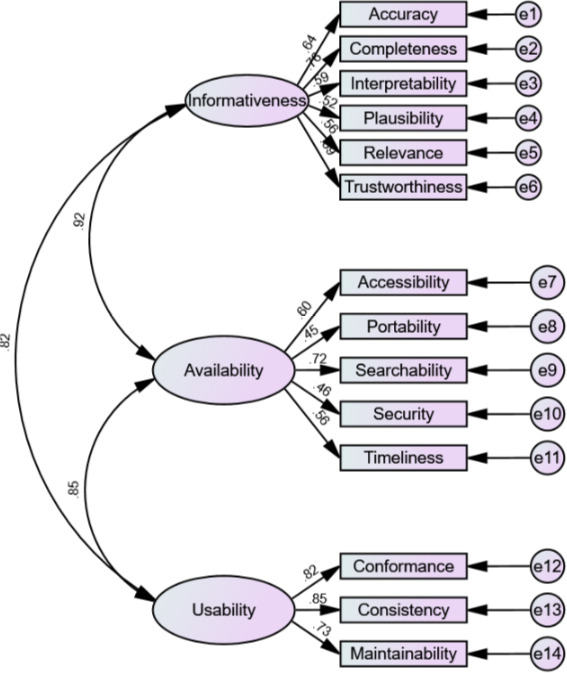
Path diagram for confirmatory factor analysis The rectangular shapes represent the observed variables (ie, information quality dimensions) that were directly measured in the survey. The circular shapes represent the latent variables (ie, information quality categories) that could be inferred from the measured variables. The abbreviation “e” represents error. The connecting arrows represent covariances, with the accompanying numbers representing their estimates.

The statistical significance of positive standardized covariance estimates for all information quality dimensions supports the placement of the dimensions in their respective category. This is further illustrated in the path diagram shown in Figure 2. The high values of estimated covariances between the latent variables and the observed variables support the construct validity of the model. Still, the high covariance estimates between the latent variables (informativeness, availability, and usability) indicate overlap of the categories.

**Table 4.** Standardized and unstandardized estimates of the covariance.

Standardized covariance estimates	Unstandardized covariance estimates	SE	Critical ratio	*P* value
Accuracy	0.64	1.00	N/A^a^	N/A	N/A
Completeness	0.76	1.18	0.18	6.41	<.001
Interpretability	0.59	0.77	0.15	5.27	<.001
Plausibility	0.52	0.53	0.11	4.72	<.001
Relevance	0.56	0.61	0.12	5.00	<.001
Trustworthiness	0.59	0.57	0.11	5.24	<.001
Accessibility	0.60	1.00	N/A	N/A	N/A
Portability	0.45	1.13	0.28	3.99	<.001
Searchability	0.72	1.43	0.25	5.66	<.001
Security	0.46	0.50	0.13	4.03	<.001
Timeliness	0.56	1.05	0.22	4.73	<.001
Conformance	0.82	1.00	N/A	N/A	N/A
Consistency	0.85	1.20	0.12	9.64	<.001
Maintainability	0.73	0.94	0.12	8.05	<.001

a N/A: not applicable.

## Discussion

### Principal Findings

The study assessed the applicability, internal consistency, and construct validity of the CLIQ framework based on the pilot CLIQ assessment of SystmOne EHRs. Overall, the CLIQ dimensions had good quality scores except for portability which had a modest score. The inter-item correlations were all positive and not likely due to chance. The Cronbach α score demonstrated a good internal consistency of the CLIQ framework and its informativeness, availability, and usability subscales. The results of the Confirmatory Factor Analysis provided a modest support for the construct validity of the CLIQ framework.

### Comparison With Previous Literature

The combination of good security and modest portability of clinical information in SystmOne indicates the possibility of a trade-off between security and portability. Portability might have been limited inadvertently to improve the security of clinical information. Similar trade-offs, such as between accessibility and security, have been documented in the literature [29]. Software developers need to be vigilant to identify potential trade-offs that may compromise the quality and safety of care.

The positive and mostly significant correlations between the items in the scale indicated a close relationship between the dimensions [30]. This was not unexpected because all dimensions are components of the same CLIQ framework. The generally low values of the correlations demonstrated that the dimensions, although related, are distinct [30]. The high correlation between conformance and consistency of presentation is understandable, as both concern presentation of information. Similarly, the high covariance between the latent variables (informativeness, availability, and usability), which indicate that the categories are not entirely distinct, is not strange in information quality research. Categories in information quality frameworks are not mutually exclusive as each dimension in an information framework often fits into multiple categories [31]. However, there is a scope for model revision to further explore this overlap in future CLIQ research.

The overall good internal consistency of the CLIQ framework and its subscales (ie, informativeness, availability, and usability) indicates the reliability of the CLIQ framework [32]. Although the study was unable to rely on fit indices primarily influenced by sample size such as the chi-square goodness-of-fit index [23], the index least affected by sample size, the SRMR [28], supports the model fit.

This study used similar methods as a study on the validation of the Modified Enlight Suite (MES), a generic mHealth assessment questionnaire [25]. The MES study demonstrated an overall good internal consistency and modest construct validity of the MES based on a survey of more than a thousand medical students and health care professionals who assessed a freely downloadable COVID-19 app in Ireland [25]. The differences in the uptakes of the 2 studies are probably related to the choice of DHTs and study population. This study population was limited to health care professionals using SystmOne. In contrast, the MES study was open to all health care professionals and medical students who could download the Irish COVID-19 app.

### Strengths and Weaknesses

To the best of our knowledge, the CLIQ framework is the first information quality framework for DHTs, of which the internal reliability and construct validity have been assessed. Only the face and content validity of most existing information quality frameworks were assessed [11]. This study went a step further to explore the internal consistency and construct validity of the CLIQ framework.

Although the choice of SystmOne EHRs ensured participation of a multidisciplinary population of health care professionals in the testing of the CLIQ framework, allied health professionals were less represented. In addition, the voluntary nature of recruitment established a potential for systemic bias because those who were engaged in digital solutions were more likely to respond. The use of mandatory responses, although useful for survey data completeness, could introduce bias if participants provided less thoughtful responses just to complete the survey. Finally, the low response rate limited the assessment of the construct validity of the CLIQ framework [24]. The low uptake was probably due to the busy schedules of the health care professionals at a time when the National Health System was under immense pressure due to COVID-19 pandemic. Nevertheless, the sample size was sufficient to assess the CLIQ framework’s applicability and reliability as demonstrated by the narrow CIs for the Cronbach α coefficients. Future studies on the validity of the CLIQ framework need to consider and address these limitations.

### Implications for Practice

#### Information Quality Assurance System

The CLIQ research highlights the importance of information quality and its relevance to the quality and safety of care. Therefore, establishing a robust system for information quality assurance in health care institutions is essential. Such an information quality assurance system entails regular checks and monitoring, data validation, and information quality audits [33]. The CLIQ framework could be a useful tool for information quality audits. The information quality assurance system should be integrated into the information governance system, where one already exists, to prevent duplication and fragmentation. Designating clinicians with additional informatics training to oversee such information quality assurance system is necessary to ensure its successful implementation because they understand the clinical and technological aspects of patient care [34]. Although health care institutions within high-income countries have designated clinical informatics roles, such as the chief clinical information officer, the job description is still evolving [35]. It is vital to both introduce these roles in health facilities and expand the responsibilities to include information quality assurance.

#### Informatics Training and Education for Health Care Professionals

The CLIQ framework demonstrates that information quality is multidimensional, and understanding its meaning, relevance, and assessment requires some training. The clinicians who participated in the international eDelphi study expressed concerns that health care professionals without informatics training might be unfamiliar with information quality–related terms [21]. In addition, information quality problems, such as missing and inaccurate information, could result from human errors [36]. Informatics training could provide health care professionals with knowledge about the meaning, relevance, and assessment of information quality. Training could also be used to address information quality problems through promotion of good practice such as proper documentation and adequate record keeping. Informatics training should be included in health care professionals’ prequalification and postqualification education competencies to also keep them up-to-date of the ever-changing information landscape in digital health [37]. However, care should be taken not to complicate an already-complex medical curriculum with extensive informatics training [37]. Rather, efforts should be made to provide appropriate level of informatics training that is commensurate with individual and institutional needs. Although informatics training could build health professionals’ competencies around information quality, mistakes will continue to occur because “to err is human” [38]. Therefore, it is vital to institute a system that reduces incidence and impact of errors.

#### Automated Error Detection and Data Validation Systems

The quality of clinical information could be improved by setting up automated error detection and data validation systems in DHTs. Machine learning and artificial intelligence algorithms could be applied for automated data validation and real-time error detection with errors flagged during data entry [39]. Automated error detection and data validation systems could reduce the likelihood of wrong data entry as well as prompt early correction of errors before patient safety is compromised. However, lack of trust in machine learning and artificial intelligence could make health care professionals ignore or override automated warnings and alerts [40].

#### Interoperability and Integration of Digital Health Systems

Interoperability enhances seamless flow of information across different DHTs such as EHRs, laboratory information system, and electronic triage system [41]. Seamless health information exchange between multiple platforms improves quality of clinical information [42]. Communication between systems enhances timely access to up-to-date information in or near real time [42]. Accuracy is enhanced when information is obtained directly from source with elimination of possible changes during transcription [41]. Portability and interoperability could be improved through adoption of clinical data models (eg, openEHR), standardized terminologies (eg, Systematized Nomenclature of Medicine Clinical Term—SNOMED CT), and clinical coding system (eg, *International Classification of Diseases, Tenth Revision*—*ICD-10*), which promote consistent presentation of clinical information [43,44]. Although interoperability of digital health systems is desirable, a huge cost is often required for its implementation. Concerns about security of information may also be an obstacle due to increased access to information [41]. Security concerns could be addressed by obtaining prior informed consent for information sharing from patients when they register with the health service.

#### User-Friendly Design Interface

The quality of data entry could be improved with a user-friendly design interface [45]. Accuracy and completeness of the information in the DHTs could be enhanced with features such as drop-down menus, prepopulation fields, and validation prompts. Faulty design interface can affect consistency of presentation and conformance. A drop down with different units of the same medication can lead to medication errors [10]. Searchability of information improves when the design interface allows smooth navigation through the digital system.

### Implications for Policy

#### Information Quality Requirements

Policies and guidelines are needed to define and communicate information quality requirements for DHTs. Policies should communicate the official position of an institution on information quality requirements while guidelines should provide clear guidance on how to meet the requirements. The CLIQ framework could be used as a conceptual guide for information quality requirements. Policies and guidelines are needed to address issues relating to information quality, including interoperability, privacy and confidentiality, information format, design interface, training, and so forth. These issues should be addressed collectively in a single information quality policy or individually with different guidelines based on the needs of the institution. It is essential to consider whether the information is used locally, nationally, or internationally. The process of formulating, implementing, and disseminating the policies needs to involve all stakeholders, as described subsequently.

#### Collaboration and Partnerships Among Information Stakeholders

The development of the CLIQ framework demonstrates the importance of multidisciplinary and international collaboration in the development of information quality standards. The CLIQ framework was developed with inputs of scores of professionals from more than 10 countries. Establishing and implementing information quality standards require collaboration among health care organizations, software developers and vendors, regulatory bodies, patient support group, professional organizations, and so forth. Collaboration will ensure buy-in of all information stakeholders and facilitate the implementation process.

#### Certification and Regulation

Enforcement of information quality standards for DHTs requires regulatory oversights. Regulatory bodies for DHTs in different countries and regions, such as the United States Food and Drug Administration and the Medicines and Healthcare products Regulatory Agency, need to include information quality requirements as part of prequalification criteria for DHTs. Information quality of DHTs should be assessed using evidence-based approach such as the CLIQ framework before they are certified for use in health care facilities. Only DHTs that meet specified prequalification criteria, including information quality requirements, should be certified by the regulatory bodies.

### Implication for Future Research

#### Evidence-Based Approach

The CLIQ framework was developed and validated using an approach that combined evidence from literature and empirical studies. We conducted a systematic review of frameworks, rather than interventions, based on the BeHeMoTh procedure [46]. The international eDelphi study obtained quantitative and qualitative evidence from clinicians across 10 countries [21]. The pilot CLIQ assessment used a systematic approach to investigate the reliability and validity of this novel tool. Overall, the evidence-based approach used while developing the CLIQ framework provides a methodological model which could be adopted in future research while developing pragmatic frameworks.

#### Information Quality Research

The systematic review of literature showed a dearth of information quality research relating to DHTs [11]. As DHTs are used more commonly worldwide, there is an increased need for information quality research to match the growth in technological innovations. The CLIQ framework demonstrates the importance of information quality research in addressing contemporary issues relating to DHTs. Some information quality dimensions, which were hitherto not included in most existing information quality frameworks for DHTs, are included in the CLIQ framework. Searchability and maintainability are more relevant due to technological advancement which has made it possible to capture, process, and store an increasing volume of digital information. Searchability, that is, locating needed information from the wide range of available information, is thus essential. Maintainability of information is also important to ensure that information quality is not sacrificed with increasing quantity of information. Similarly, portability has become more relevant than ever with the current tendency toward integration and interoperability of DHTs.

#### Patient Perspective on Information Quality

This study focused on health care professionals as end users of clinical information in DHTs. However, patients have also become end users of clinical information in DHTs. Patients are increasingly viewing and contributing more information into DHTs, especially since the COVID-19 pandemic [47]. Multiple DHTs, such as AskmyGp (Evergreen Health Solution Ltd), eConsult (eConsult Health Ltd), and Accurx (Accurx Ltd), allow patients to enter text using their own words and upload photographs that could be saved in the EHRs. In addition, many wearable devices and mobile apps generate consumer health data, nowadays, that could be imported into the EHRs. It would therefore be useful to explore the patients’ perspective on information quality in future studies and incorporate their views and suggestions into the CLIQ framework.

### Conclusions

The CLIQ research highlights the importance of information quality and its relevance to the quality and safety of care. The CLIQ framework demonstrated a high reliability and a modest construct validity. The CLIQ framework offers a pragmatic approach to assessing the quality of clinical information in DHTs and could be applied as part of information quality assurance systems in health care settings to improve quality of health information.

## Supplementary material

10.2196/58125 Multimedia Appendix 1 Clinical Information Quality (CLIQ) questionnaire.

10.2196/58125 Multimedia Appendix 2 Inter-item correlation matrix.

10.2196/58125 Checklist 1 Checklist for Reporting Results of Internet E-Surveys.

## Abbreviations

CFI: comparative fit index
CHERRIES: Checklist for Reporting Results of Internet E-Surveys
CLIQ: clinical information quality
DHT: digital health technology
EHR: electronic health record
MES: modified enlight suite
SRMR: Standardized Root Mean Square Residual
