# Supplementary material for: A Novel Framework to Assess Clinical Information in Digital Health Technologies: Cross-Sectional Survey Study
Source: JMIR Med Inform. 2025 May 30;13:e58125. doi: 10.2196/58125 (PMC12143851; doi:10.2196/58125)
Supplement: Multimedia Appendix 1 [file medinform-v13-e58125-s001.pdf]

Clinical Information Quality (CLIQ) Assessment Questionnaire

Digital Health Technology…………………………………….

Date of Assessment…………………………………………….

For each of the attributes (e.g., accuracy), please select which of the three options (e.g., accurate, partly accurate and inaccurate) best describes clinical information in the digital health technology you are assessing based on your personal clinical experience.

Accuracy: Is the information accurate?

☐ **Accurate**. The information is accurate.

☐ **Partly accurate**: The digital health technology contains some inaccurate information which is unlikely to compromise patient safety or quality of care (i.e., unlikely to cause death, harms or disruption/delay in patient care).

☐ **Inaccurate**: The digital health technology contains some inaccurate information which is likely to compromise patient safety or quality of care (i.e., likely to cause death, harms or disruption/delay in patient care).

Completeness: Is any information missing?

☐ **Complete**: No information reasonably expected to be documented/recorded is missing (e.g., name, dose and indication of medication prescribed).

☐ **Partly complete**: Information reasonably expected to be documented/recorded is partially recorded (e.g., name of medication recorded without the dose).

☐ **Absent**: Information reasonably expected to be documented is absent/missing (e.g., name, dose and indication of medication missing).

Interpretability: Can the information be interpreted?

☐ **Interpretable**: The information can be interpreted without a need for additional resources (e.g., reference range included alongside laboratory results).

☐ **Partly interpretable**: The information can be interpreted only with additional resources or assistance (e.g., reference materials required because reference range not included with laboratory results).

☐ **Uninterpretable**: The information cannot be interpreted even with additional resources (e.g., unable to interpret laboratory results even after consulting reference materials).

Plausibility. Is the information credible, reasonable, or likely?

☐ **Plausible**. Is the information credible, reasonable, or likely/probable (e.g., fever in a child with sepsis).

☐ **Partly plausible**. The information is probable only in exceptional circumstances (e.g., normal body temperature in a child with sepsis).

☐ **Implausible**: The information is unlikely to be true (e.g., body temperature of 100 degrees Celsius which is not compatible with life).

Trustworthiness: Is the source of the information trustworthy and verifiable?

☐ **Trustworthy**: The information is from a trustworthy source (e.g., WHO, government agencies, academic institutions, healthcare institutions, peer-reviewed medical journals).

☐ **Partly trustworthy**: The information is only partly from a verifiable source (e.g., some claims are without evidence-base).

☐ **Untrustworthy**: The information is from unverifiable and non-peer reviewed sources (e.g., social media).

Relevance: Is the information relevant to patient care?

☐ **Relevant**: The information is useful, directly connected with or related to patient care (e.g., useful for diagnosis, monitoring and treatment).

☐ **Partly relevant**: The information is useful, directly connected with or related to some aspects of patient care (e.g., useful only for discharge planning).

☐ **Irrelevant**: The information is not useful, directly connected with or related to any aspect of patient care.

Accessibility: Can the information be obtained or reached easily?

☐ **Accessible**: The information can be easily obtained at the point of care (e.g., immediate access with username and password).

☐ **Partly accessible**: The information can be obtained with some difficulties (e.g., delayed access due to frequent downtime).

☐ **Inaccessible**: The information is not easily obtainable (e.g., access requires phone calls to IT Department).

Portability: Is the information easily moved or transferred? Portability is a feature of information that allows a user to take information from one system or part of the system and transfer or "port" it to another part of the system or elsewhere (e.g., community, primary, secondary, tertiary care).

☐ **Portable**: The information is easily moved or transferred (e.g., transferable at the point of care by clinician).

☐ **Partly portable**: The information is transferable partly or transfer requires additional effort (e.g., conversion of values).

☐ **Unportable**: The information is not transferable to other system or other part of the system (e.g., requires printing).

Searchability: Can the information be easily searched for or retrieved?

☐ **Searchable**: The information is easily searched for or retrieved at the point of care.

☐ **Partly Searchable**: The information can be searched for or retrieved at the point of care with some difficulty.

☐ **Not Searchable**: The information cannot be easily searched for or retrieved at the point of care.

Security: Is the information protected from unauthorized access, corruption, damage and loss throughout its lifecycle across all applications and platforms?

☐ **Secure**: The information is protected from unauthorized access, corruption, damage and loss using a comprehensive strategy (this includes but not limited to two-factor authentication, password and smart card, role-based access, encryption, single-sign-on authentication).

☐ **Partly secure**: The information is protected from unauthorized access, corruption, damage and loss by only some rather than comprehensive strategy (e.g., requires only username and password but not role-based access).

☐ **Insecure**: The information is not protected from unauthorized access, corruption and loss (e.g., information obtainable from the hospital system using a generic account).

Timeliness: Is the information constantly updated?

☐ **Timely**: The information is constantly updated (e.g., laboratory results available in real time or near real time).

☐ **Partly timely**: There is delay in new information flowing into the system (e.g., delay in new laboratory results becoming available at the point of care).

☐ **Non-current**: The information is outdated (e. g., current laboratory results not available at the point of care).

Conformance: Is the information presented in a format that complies with national standards/guidelines?

Note: Presentation format includes but not limited to units (e.g., Celsius, Fahrenheit), structure (e.g., drop down, free text), scoring system (e.g., GCS- Glasgow Comma Scale). abbreviation (Hb vs Haemoglobin).

☐ **Conformant**: the information complies with national standards/guidelines (e.g., presentation of level of consciousness using GCS stating all the components).

☐ **Partly conformant**: the information partly complies with national standards/guidelines (e.g., presentation of level of consciousness using GCS stating only the total score).

☐ **Non-conformant**: the information does not comply with national standards/guidelines (e.g., presentation of level of consciousness not using GCS or any other recommended scoring system).

Consistency of presentation. Does the presentation of information adhere to the same set of principles / consistently in the same format?

Note: Presentation format includes but not limited to units (e.g., Celsius, Fahrenheit), structure (e.g., drop down, free text), scoring system (e.g., GCS- Glasgow Comma Scale). abbreviation (Hb vs Haemoglobin).

☐ **Consistently presented**: The information is presented consistently in the same format (e.g., Adrenaline dose consistently expressed in mg across the system).

☐ **Consistently presented partly**: The information is presented in multiple formats in different parts of the system (Adrenalin dose presented as mg and ml in different parts of the system).

☐ **Inconsistently presented**: The information is presented in multiple formats in the same part of the system (Adrenalin dose presented as mg and ml within the same drop-down).

Maintainability: Can the information be easily maintained (e.g., corrected, updated, upgraded, adapted) to achieve intended improvement?

☐ **Maintainable**: The information can be maintained without any difficulties (e.g., immediate correction of wrong entry in electronic health records automatically approved with audit trail).

☐ **Partly maintainable**: The information can be maintained with resolvable difficulties (e.g., correction of wrong entry in the electronic health records requires authorization by another professional who is not its author).

☐ **Unmaintainable**: The information cannot be maintained (e.g., wrong entry cannot be corrected but requires additional entry such as an addendum to explain the modification
