# Supplementary material for: A Novel Framework to Assess Clinical Information in Digital Health Technologies: Cross-Sectional Survey Study
Source: JMIR Med Inform. 2025 May 30;13:e58125. doi: 10.2196/58125 (PMC12143851; doi:10.2196/58125)
Supplement: Multimedia Appendix 2 [file medinform-v13-e58125-s002.docx]

**Table S1: Inter-items Correlation Matrix**

|  | Accuracy | Completeness | Interpretability | Plausibility | Relevance | Trustworthiness | Accessibility | Portability | Searchability | Security | Timeliness | Conformance | Consistency | Maintainability |
| --- | --- | --- | --- | --- | --- | --- | --- | --- | --- | --- | --- | --- | --- | --- |
| Accuracy  *P*-value | 1.000 | .555 | .356 | .198 | .377 | .336 | .375 | .240 | .443 | .231 | .378 | .465 | .485 | .382 |
|  |  | <.001 | <.001 | .039 | <.001 | <.001 | <.001 | .012 | <.001 | .015 | <.001 | <.001 | <.001 | <.001 |
| Completeness  *P*-value | .555^**^ | 1.000 | .463 | .348 | .405 | .434 | .394 | .248 | .498 | .413 | .507 | .488 | .519 | .529 |
|  | <.001 |  | <.001 | <.001 | <.001 | <.001 | <.001 | .009 | <.001 | <.001 | <.001 | <.001 | <.001 | <.001 |
| Interpretability  *P*-value | .356^**^ | .463^**^ | 1.000 | .333^**^ | .272 | .320 | .284 | .216 | .359 | .403 | .339 | .650 | .513 | .387 |
|  | <.001 | <.001 |  | <.001 | .004 | <.001 | .003 | .024 | <.001 | <.001 | <.001 | <.001 | <.001 | <.001 |
| Plausibility  *P*-value | .198^*^ | .348^**^ | .333^**^ | 1.000 | .450 | .562 | .218 | .078 | .308 | .334 | .270 | .283 | .338 | .379 |
|  | .039 | <.001 | <.001 |  | <.001 | <.001 | .023 | .422 | .001 | <.001 | .005 | .003 | <.001 | <.001 |
| Relevance  *P*-value | .377^**^ | .405^**^ | .272^**^ | .450^**^ | 1.000 | .430 | .416 | .130 | .356 | .288 | .297 | .269 | .287 | .441 |
|  | <.001 | <.001 | .004 | <.001 |  | <.001 | <.001 | .179 | <.001 | .002 | .002 | .005 | .002 | <.001 |
| Trustworthiness  *P*-value | .336^**^ | .434^**^ | .320^**^ | .562^**^ | .430^**^ | 1.000 | .321 | .154 | .300 | .455 | .342 | .260 | .390 | .354 |
|  | <.001 | <.001 | <.001 | <.001 | <.001 |  | <.001 | .109 | .002 | <.001 | <.001 | .006 | <.001 | <.001 |
| Accessibility  *P*-value | .375^**^ | .394^**^ | .284^**^ | .218^*^ | .416^**^ | .321^**^ | 1.000 | .410 | .422 | .219 | .260 | .388 | .401 | .352 |
|  | <.001 | <.001 | .003 | .023 | <.001 | <.001 |  | <.001 | <.001 | .022 | .006 | <.001 | <.001 | <.001 |
| Portability  *P*-value | .240^*^ | .248^**^ | .216^*^ | .078 | .130 | .154 | .410^**^ | 1.000 | .443 | .041 | .356 | .296 | .361 | .387 |
|  | .012 | .009 | .024 | .422 | .179 | .109 | <.001 |  | <.001 | .668 | <.001 | .002 | <.001 | <.001 |
| Searchability  *P*-value | .443^**^ | .498^**^ | .359^**^ | .308^**^ | .356^**^ | .300^**^ | .422^**^ | .443^**^ | 1.000 | .275 | .348 | .468 | .456 | .604 |
|  | <.001 | <.001 | <.001 | .001 | <.001 | .002 | <.001 | <.001 |  | .004 | <.001 | <.001 | <.001 | <.001 |
| Security  *P*-value | .231^*^ | .413^**^ | .403^**^ | .334^**^ | .288^**^ | .455^**^ | .219^*^ | .041 | .275^**^ | 1.000 | .300 | .323 | .391 | .376 |
|  | .015 | <.001 | <.001 | <.001 | .002 | <.001 | .022 | .668 | .004 |  | .002 | <.001 | <.001 | <.001 |
| Timeliness  *P*-value | .378^**^ | .507^**^ | .339^**^ | .270^**^ | .297^**^ | .342^**^ | .260^**^ | .356^**^ | .348^**^ | .300^**^ | 1.000 | .345 | .379 | .464 |
|  | <.001 | <.001 | <.001 | .005 | .002 | <.001 | .006 | <.001 | <.001 | .002 |  | <.001 | <.001 | <.001 |
| Conformance  *P*-value | .465^**^ | .488^**^ | .650^**^ | .283^**^ | .269^**^ | .260^**^ | .388^**^ | .296^**^ | .468^**^ | .323^**^ | .345^**^ | 1.000 | .751 | .518 |
|  | <.001 | <.001 | <.001 | .003 | .005 | .006 | <.001 | .002 | <.001 | <.001 | <.001 |  | <.001 | <.001 |
| Consistency  *P*-value | .485^**^ | .519^**^ | .513^**^ | .338^**^ | .287^**^ | .390^**^ | .401^**^ | .361^**^ | .456^**^ | .391^**^ | .379^**^ | .751^**^ | 1.000 | .561 |
|  | <.001 | <.001 | <.001 | <.001 | .002 | <.001 | <.001 | <.001 | <.001 | <.001 | <.001 | <.001 |  | <.001 |
| Maintainability  *P*-value | .382^**^ | .529^**^ | .387^**^ | .379^**^ | .441^**^ | .354^**^ | .352^**^ | .387^**^ | .604^**^ | .376^**^ | .464^**^ | .518^**^ | .561^**^ | 1.000 |
|  | <.001 | <.001 | <.001 | <.001 | <.001 | <.001 | <.001 | <.001 | <.001 | <.001 | <.001 | <.001 | <.001 |  |
